# Probiotic *Bifidobacterium longum* Subsp. *longum* 5^1A^ Protects Mice from Genotoxic and Metabolic Alterations Induced by Subchronic Exposure to a Low-Dose Pesticide Cocktail

**DOI:** 10.1021/acsomega.5c04121

**Published:** 2025-08-04

**Authors:** Mirna Maciel d’Auriol Souza, Viviani Mendes de Almeida, Clênio Silva Cruz, Victor de Melo Rocha, Paola Caroline Lacerda Leocárdio, Sumaia Araújo Pires, Maysa do Vale Oliveira, Tainá Brumate, Maria José Nunes de Paiva, Jacqueline Isaura Alvarez-Leite, Geovanni Dantas Cassali, Flaviano Santos Martins, Angélica Thomaz Vieira, Leiliane Coelho André

**Affiliations:** † Laboratório de Análises Toxicológicas (LATO), Faculdade de Farmácia, Universidade Federal de Minas Gerais institution, Belo Horizonte 31270-901, Brazil; ‡ Laboratório de Imunomodulação (LMI), Instituto de Ciências Biológicas, 28114 Universidade Federal de Minas Gerais, Belo Horizonte 31270-901, Brazil; § Laboratório de Lipídeos, Aterosclerose e Bioquímica Nutricional (LABIN), Instituto de Ciências Biológicas, Universidade Federal de Minas Gerais, Belo Horizonte 31270-901, Brazil; ∥ Departamento de Ciências da Saúde (DCS), Universidade Federal do Espírito Santo, Vitória, São Mateus 29945500, Brazil; ⊥ Laboratório de Patologia Comparada (LPC), Instituto de Ciências Biológicas, 28114 Universidade Federal de Minas Gerais, Belo Horizonte 31270-901, Brazil; # Laboratório de Agentes Bioterapêuticos (LABio), Instituto de Ciências Biológicas, 28114 Universidade Federal de Minas Gerais, Belo Horizonte 31270-901, Brazil

## Abstract

Environmental exposure to low levels of pesticide mixtures through food and domestic settings raises significant public health concerns due to their potential link to metabolic diseases, including obesity, type 2 diabetes, and cancer. As the intestinal microbiota and gastrointestinal tract serve as primary contact points for these contaminants, potentially eliminating residues or suffering direct damage leading to dysbiosis, probiotic interventions have emerged as promising therapeutic strategies for metabolic disorders. This study aimed to evaluate, in mice, the consequences of subchronic exposure to low doses of a mixture of three pesticides: glyphosate (15 mg/kg body weight/day), imidacloprid (5 mg/kg body weight/day), and tebuconazole (4 mg/kg body weight/day). Mice were exposed for 6 weeks. The probiotic *Bifidobacterium longum* subsp. *longum* strain 5^1A^ was employed as a therapeutic strategy for gut microbiota modulation. Exposed animals experienced adverse metabolic and genotoxic effects including increased cholesterol and reduced insulin sensitivity, oxidative stress, and intestinal dysbiosis. *B. longum* 5^1A^ reversed these effects, improving metabolism and reducing prediabetes markers. As the first study examining this specific pesticide combination, imidacloprid, tebuconazole, and glyphosate highlight the distinct impact of pesticide mixtures on health, suggesting that intestinal dysbiosis may drive the observed metabolic changes, with microbiota modulation aiding recovery.

## Graphical abstract

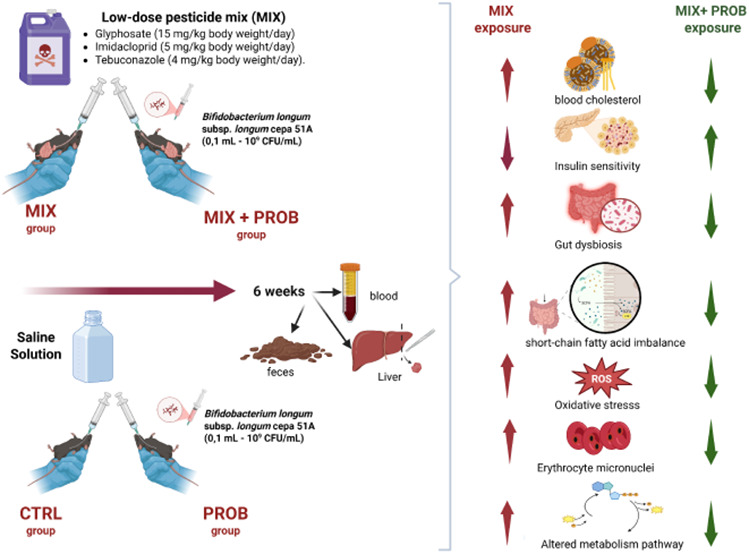

## 1 Introduction

Due to the extensive and continuous use of pesticides in various activities, such as agriculture and public health campaigns, their use can affect human health and the environment. Environmental exposure to pesticides and their residues causes several harmful effects directly related to the kind of pesticide, frequency, and duration of exposure, as well as the amount of product.^1^ The potential long-term risk to humans is associated with inflammation, oxidative stress, and intestinal disorders, and, consequently, metabolic diseases, such as type 2 diabetes, obesity, and cancer.^2–4^

The multiresidue pesticides in foods are the most important source of environmental human exposure. However, it is difficult to predict the type of additive, synergistic, or antagonistic interaction (these chemical substances can have on an organism).^5^ Glyphosate (GLY—herbicide), imidacloprid (IMI—insecticide), and tebuconazole (TEB—fungicide) are the three most commercialized pesticides detected in foods in several countries, including Brazil.^6–9^ There are no studies in the literature that show the toxic effects of this mixture. However, we identified many reports of their individual adverse effects in low doses or other mixtures associated with disturbing the homeostasis of nontarget organisms, insulin resistance, and obesity,^10–13^ oxidative stress, and genotoxic effects.^6,7,14,15^

The gastrointestinal tract is the principal pathway for contact with contaminants present in food. Consequently, the intestinal microbiota are the first to metabolize these substances, even before the liver. It is estimated that more than 1300 environmental pollutants can be metabolized by the intestinal microbiota, which promotes biotransformation through a series of reactions such as reduction, lyase reaction, functional group transfer, hydrolysis, and enzymatic transformation.^16^ The intestinal microbiota are crucial and influence the health of the host. The increased use of pesticides, for example, can disrupt its homeostasis (dysbiosis) by altering its composition and, consequently, affecting its metabolites.^17^

*Bifidobacterium longum* 5^1A^ is a probiotic strain that was isolated from the feces of a healthy 5-year-old child from Salvador, Bahia, Brazil. It has 26 unique genes related to carbohydrate metabolism and acetate production, offering more benefits to the host’s health reported in several studies.^18–25^

In environmental toxicology, metabolomics is used to identify new biomarkers of chemical contaminants. It can clarify these agents’ toxicity mechanisms, allowing for the study of altered metabolic pathways.^26^ The metabolomic analysis measures metabolites related to biological processes and provides information about the organism’s interaction with the environment.^27^ Analysis of the metabolome of animals exposed to the pesticides IMI, TEB, and GLY identified that they can alter energy metabolism,^28,29^ galactose,^28,30^ purines, urea,^28^ glutathione,^31^ pentose phosphate,^29,31^ pyruvate, nucleotides,^29^ lipid, arachidonic acid pathways, biosynthesis of steroid hormones,^32^ and amino acids.^28,32^

Human exposure to pesticides is due to a mixture of chemicals in a meal rather than a single compound. Evaluating a mixture of pesticides at low doses is more complex because of possible additive, synergistic, or antagonistic effects. This study evaluated the homeostasis, genotoxicity, and metabolome of female C57bl/6 mice by oral exposure at low doses to a mixture of GLY, IMI, and TEB for 6 weeks. At the same time, it evaluated the effect of the probiotic *B. longum* 5^1A^ in preventing possible damage caused by the mixture. This is the first study that evaluates the mixture of these three pesticides associated with metabolomic analysis and intestinal microbiota.

## 2 Materials and Methods

### 2.1 Animals and Exposure Model

Twenty-four female C57bl/6 mice (age: 6 weeks; specific pathogen-free, SPF) from the Central Animal Facility of the Federal University of Minas Gerais (BC/UFMG) were kept in cages (floor area: 451 cm^2^) (Alesco Ind. e Com. Ltd.a, Campinas, SP, Brazil), with a 12 h day/night cycle and controlled aeration and temperature (23 °C). Access to filtered water and solid food was ad libitum. The mice were fed a balanced diet containing corn, soy, rice, and wheat (Presence Labina, standard chow, Brazil), providing all necessary nutrients.

The exclusive use of female mice in this initial investigation was driven by the imperative to address sex-specific responses to pesticide mixtures, acknowledging that both sexes are exposed in real-world scenarios. Future studies will extend these investigations to male cohorts to provide a comprehensive understanding of these toxic effects.

The animals were randomly divided into four groups (six animals per group). The groups that received the pesticide mixture were identified as MIX; the groups that received treatment with probiotics were identified as PROB, the group that received pesticides and probiotics was identified as MIX+PROB, and the group that received water and saline without pesticides and probiotics was identified as CTRL. The treatments lasted 6 weeks (subchronic exposure, according to OECD 408 – Guideline for the Testing of Chemicals).^33^ The animals were weighed individually from 10 days before the start of the experiment (acclimation) until the day of euthanasia (day 42) once a week.

The experimental procedures in this study were conducted in accordance with the ethical principles of animal experimentation adopted by the Ethics Committee on the Use of Animals of the Federal University of Minas Gerais (CEUA/UFMG), approved under protocol n° 123/2022.

### 2.2 Chemicals

All standards (Imidacloprid 98.5% Dr Ehrenstorfer GmbH, CAS number 138261–41–3; Tebuconazole 99.3% Pestanal Analytical Standard, Sigma-Aldrich, CAS number 107534–96–3; and Glyphosate 98.1% Pestanal Analytical Standard, Sigma-Aldrich, CAS number 1071–83–6), and reagents used were of analytical grade. The purified water was obtained using a Direct-Q3 UV system (Milli-Q, Merck, Germany) with a specific resistivity of 18.2 MΩ cm at 25 °C.

The solution of pesticides was administered once a day at doses of IMI 5 mg/kg B.W./day (100 × ADI: 0.05 mg/kg B.W./day), TEB 4 mg/kg B.W./day (133 × ADI: 0.03 mg/kg B.W./day), and GLY 15 mg/kg B.W./day (30 × ADI: 0.5 mg/kg B.W./day). The safety doses (Acceptable Daily Intake, ADI) were referenced by ANVISA (National Health Surveillance Agency, Brazil), and all doses in the study are below the NOAEL doses.^34^

### 2.3 Probiotic *B. longum* 5^1A^

The probiotic (PROB) *B. longum* subsp. *longum* strain 5^1A^, belonging to the Laboratory of Biotherapeutic Agents (UFMG) culture collection, was cultivated in De Man, Rogosa, and Sharpe (MRS) broth at 37 °C for 48 h under anaerobic conditions without agitation. The anaerobic environment was obtained using a chamber (Forma Scientific Company, Marietta, OH, USA) with an atmosphere containing 80% N_2_, 10% H_2_, and 10% CO_2_.^21^ The probiotic refills’ validity was 2 weeks to maintain the probiotic’s genetic integrity. Before the expiration date, a new aliquot of the original probiotic (stock at −80 °C) was thawed and prepared as described above.

### 2.4 Experimental Design

Each animal in the MIX and MIX+PROB groups received 0.1 mL of the mixture by gavage in the afternoon. Animals in the CTRL and PROB groups received only purified water. The mixture was prepared daily to avoid the degradation of the pesticides. The animals (PROB and MIX+PROB groups) were treated with an aliquot of 0.1 mL of the probiotic (approximately 10^9^ log CFU/mL of *B. longum* 5^1A^ bacteria) in sterile 0.9% saline, every morning, respecting the circadian cycle of animals. The CTRL and MIX groups received only 0.1 mL of sterile, 0.9% saline.

At the end of the study period (6 weeks), the mice were anesthetized (80 mg/kg ketamine and 10 mg/kg xylazine) and euthanized by exsanguination, followed by cervical dislocation. Plasma samples were prepared by centrifugation (2,000 *g*; 10 min; 4 °C) and stored at −80 °C until analysis.

### 2.5 Clinical Evaluation and Biochemical Parameters in Blood

The relative weight of the liver and adipose tissue is calculated using the following equation: organ weight × 100/body weight. The final result is expressed as a percentage (%).

Blood glucose was determined using a glucometer (Roche, model: Accu-Chek Active). The result was expressed in mg of glucose/dL for fasting glucose using the method described by Han^35^ and for the insulin sensitivity test using the method described by Santos.^36^

Total cholesterol and triglyceride in serum were measured after 6 weeks of pesticide exposure using an analysis kit from Bioclin, Brazil, with a spectrophotometer at 500 nm, and concentrations are expressed in mg/dL.

### 2.6 Untargeted-Metabolomics

The metabolomic analysis was performed on a serum sample prepared as described previously by Rey-Stolle.^37^ The quality control samples (QCs) were analyzed at the beginning, at the end, and periodically throughout the assay to evaluate the stability of analytical performance. Samples were analyzed randomly.

The chromatographic conditions were standardized according to the protocol by Fiehn^27^ using a gas chromatograph system (model 7890A) coupled to a mass spectrometer quadrupole detector (model 5975C) from Agilent Technologies. The data were acquired by using Agilent MassHunter Workstation Data Acquisition software. Software Agilent ChemStation and Mass Profiler Professional – MPP (Agilent Technologies) and Automated Mass Spectral Deconvolution and Identification System – AMDIS (https://www.amdis.net) were used for spectral deconvolution, alignment, and data filtering. Data normalization, scaling, transformation, and possible metabolic pathways were performed in MetaboAnalyst version 6.0 (https://www.metaboanalyst.ca/).

The Sum method was used to normalize the data, the log transformation was applied to transform heteroscedastic data, and Auto Scaling was used to scale the model equally. PCA was used for the exploratory analysis of serum to provide a simple global visualization of the data and evaluate the analytical stability, quality, and reliability of the obtained data. PLS-DA, without QCs, was processed like PCA (Sum, log transformation, and Auto Scaling). A VIP (Variable Importance in Projection) greater than 1 was used to identify essential metabolites. *R* ^2^ > 0.7 and *Q* ^2^ > 0.4 were considered adequate for validating the model.

Statistical analyses were performed using GraphPad Prism version v8 (GraphPad Software, San Diego, CA, USA). After analyzing normality (Shapiro-Wilk) and the presence of outliers (HOUT), the Mann–Whitney test or Wilcoxon test with Dunn correction (post hoc) was used (nonparametric data). The two-way ANOVA test for repeated measures was used to analyze body weight. A *p*-value < 0.05 was considered statistically significant.

### 2.7 Fecal Microbiota Cultivable Analysis

Two days before euthanasia, the animals’ feces were aseptically collected in a sterile 2 mL plastic tube under laminar flow, weighed, and solubilized (1:10) in sterile 0.9% w/v saline solution.^38^

Plates with selective and differential media were adopted to cultivate fecal microbiota populations as follows: blood agar was used for total aerobic microbiota CFU count, and blood agar supplemented with hemin and menadione 1% v/v was used for total anaerobic microbiota CFU count. Mannitol Salt agar was used for the isolation of *Staphylococcus*; MacConkey agar for the isolation of *Enterobacteriaceae*; Bile Esculin agar for the isolation of *Streptococcus* and *Enterococcus*; *Bacteroides* Bile Esculin agar added with 0.1% v/v gentamicin (BBE) for the isolation of *Bacteroides*; De Man, Rogosa, and Sharpe (MRS) agar in aerobiosis for the isolation of lactic acid bacteria (LAB), and in anaerobiosis for the isolation of *Bifidobacterium* and LAB.

The plates were incubated under aerobic and anaerobic conditions per the needs of the microorganism of interest, at 37 °C for 24 and 48h. After the incubation period, colony-forming units (CFU) were counted, and the results were expressed as the Log10 mean ± SEM of CFU/mg of feces.

### 2.8 Determination of Short Chain Fatty Acids in Feces

The Short Chain Fatty Acids (SCFAs) were analyzed in feces samples by high-performance liquid chromatography (HPLC) with UV–vis from Thermo (Finnigan Surveyor Plus). Mendes de Almeida^38^ conducted the sample preparation and chromatographic analysis. The column used was a SUPELCOGEL C610H ion exchange guard column, 6% cross-linked HPLC, 9 μm particle, 5 cm width × 4.6 mm I.D. (Sigma-Aldrich).

### 2.9 Determination of Oxidative Stress in Liver Tissue

The oxidative stress was evaluated by measuring malondialdehyde (MDA) and hydroperoxides. To normalize the results, the values were corrected by the total protein content of the tissue.^39^

Buege^40^ previously described quantifying MDA as a thiobarbituric acid reactive substance (TBARS) using a spectrophotometer at 535 nm from Celer Biotechnology S.A. (model Polaris). The TBARS determination was expressed in μmol of MDA/mg protein, calculated from the molar extinction coefficient of 1.569 × 105 m^–1^ cm^–1^.

Banerjee^41^ previously described the determination of hydroperoxides using a spectrophotometer from Celer Biotechnology S.A. (model Polaris). The result is expressed in μmol of hydroperoxides per mg proteins.

### 2.10 Micronucleus Assay in Blood

The micronucleus (MN) test was used to evaluate the genotoxic susceptibility. MN counting was performed following the criteria described by Heddle^42^ and Titenko-Holland^43^ and recommended by the OECD 474 protocol,^44^ considering for counting only intact cells with a rounded shape and intact cytoplasm. One thousand erythrocytes were counted per slide, in duplicate per animal, and the result was expressed as a frequency of MN/1000 erythrocytes. Immature and mature erythrocytes were not differentiated because the treatment in the animals was continuous, exceeding the lifespan of the erythrocyte in the mouse (4 weeks), and the splenic depletion of micronucleated erythrocytes in mice is not strong enough to compromise the detection of MN.^44^

## 3 Results

### 3.1 Subchronic Exposure to a Mixture of Pesticides Induces an Increase in Total Cholesterol, a Reduction in Triglycerides, and Insulin Resistance in Mice

The pesticide-exposed mice evidenced mild alterations in clinical parameters. Body weight (initial weight: 16.90 ± 1.033 g, mean ± SD) was used to calculate pesticide doses and evaluate physiological status, with >10% loss considered acute intoxication. As shown in Figure 1A, no statistical difference in weight trajectory emerged between CTRL and MIX groups (*p* > 0.05), and weight gain progressed normally with aging. The absence of diarrhea or significant weight deviation suggests that the mixture induced subclinical toxicity without overt distress.

**Figure 1:**
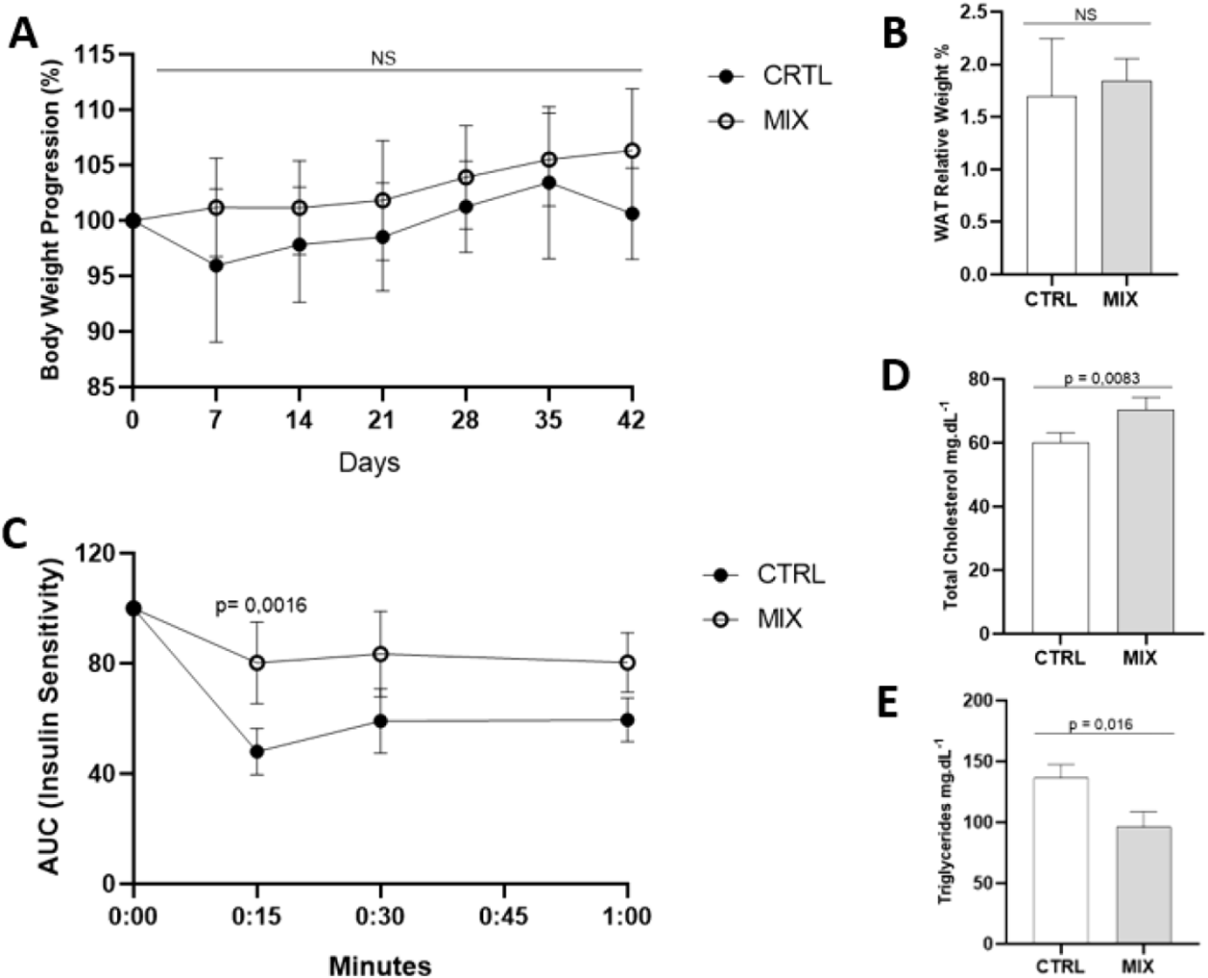
Metabolic effects of subchronic pesticide mixture exposure in C57BL/6 mice. (A) Body weight (BW) trajectory during 6-week exposure to MIX (GLY-IMI-TEB mixture, black circles) versus CTRL (vehicle control, white circles). (B) Periuterine white adipose tissue (WAT) mass normalized to body weight. (C) Glucose tolerance test showing blood glucose area under the curve (AUC) at 0, 15, 30, and 60 min postinsulin injection (MIX: white circles; CTRL: black circles). (D,E) Serum lipid profiles: (D) total cholesterol and (E) triglycerides after 6-week exposure. Data represent mean ± SD (*n* = 5 or 6/group); ns = not significant (*p* ≥ 0.05, Mann–Whitney test). All pesticide doses were below individual NOAELs.

After euthanasia, the relative weight of the periuterine WAT was evaluated (Figure 1B). There was no statistical difference between the groups (CTRL and MIX) for the relative weight data of the tissue evaluated.

Samples were collected at 3 and 6 weeks of exposure to evaluate the fasting blood glucose levels of nonexposed and exposed animals. Blood glucose did not reveal any significant differences between exposed mice and unexposed mice at 3 or 6 weeks of age (CTRL: 136.50 ± 17.01 and 139.16 ± 15.29 mg dL^–1^; MIX: 136.50 ± 23.31 and 133.30 ± 16.70 mg dL^–1^; 3 and 6 weeks, respectively). There was also no difference when each mouse was evaluated individually in paired statistical analysis. In other words, the animals showed no change in fasting blood glucose during all exposures over time.

As the animals were not fasting, statistical tests were performed on normalized data (initial value equivalent to 100%) to analyze insulin sensitivity. The MIX group showed a higher area under the curve (AUC) value than the CTRL group (Figure 1C), indicating greater sensitivity to insulin (*p* = 0.004). Delta calculated can identify the recovery of the animals after each period. The evaluation of the delta from times 0–15 min showed statistically significant results (*p* = 0.0016). The control group evidenced a reduction of 51.96 ± 8.40% in circulating glucose after 15 min of insulin injection, while a minor reduction in serum glucose corresponding to 19.71 ± 14.80% was observed in the group exposed to the mixture.

Total cholesterol and triglycerides in the MIX group compared to the CTRL group revealed significant differences (Figure 1D,E). The MIX group showed a statistically significant reduction in serum triglycerides (*p* = 0.016), with values of 136.559 ± 10.644 mg dL^–1^ for the CTRL group and 97.205 ± 13.720 mg dL ^–1^ for the MIX group. The evaluation of total cholesterol revealed the opposite result, with a serum increase in this biochemical parameter (*p* = 0.0083), presenting values of 60.094 ± 3.103 mg dL^–1^ for the CTRL group and 70.391 ± 3.890 mg dL ^–1^ for the MIX group.

### 3.2 Subchronic Exposure to a Mixture of Pesticides Alters the Metabolism of Mice after 6 Weeks

The metabolic profile of mice exposed to the pesticide mixture was significantly altered compared with controls, as demonstrated by both supervised and unsupervised analyses. The PLS-DA model (Figure 2A) showed clear group separation (*Q* ^2^ = 0.49149, *R* ^2^ *Y* = 1.00, accuracy = 0.9), with further validation of model robustness provided by Random Forest analysis (OOB error = 0.20; Figure S1). These complementary approaches confirm the reliability of the metabolic differences observed between the control and pesticide-exposed groups.

**Figure 2:**
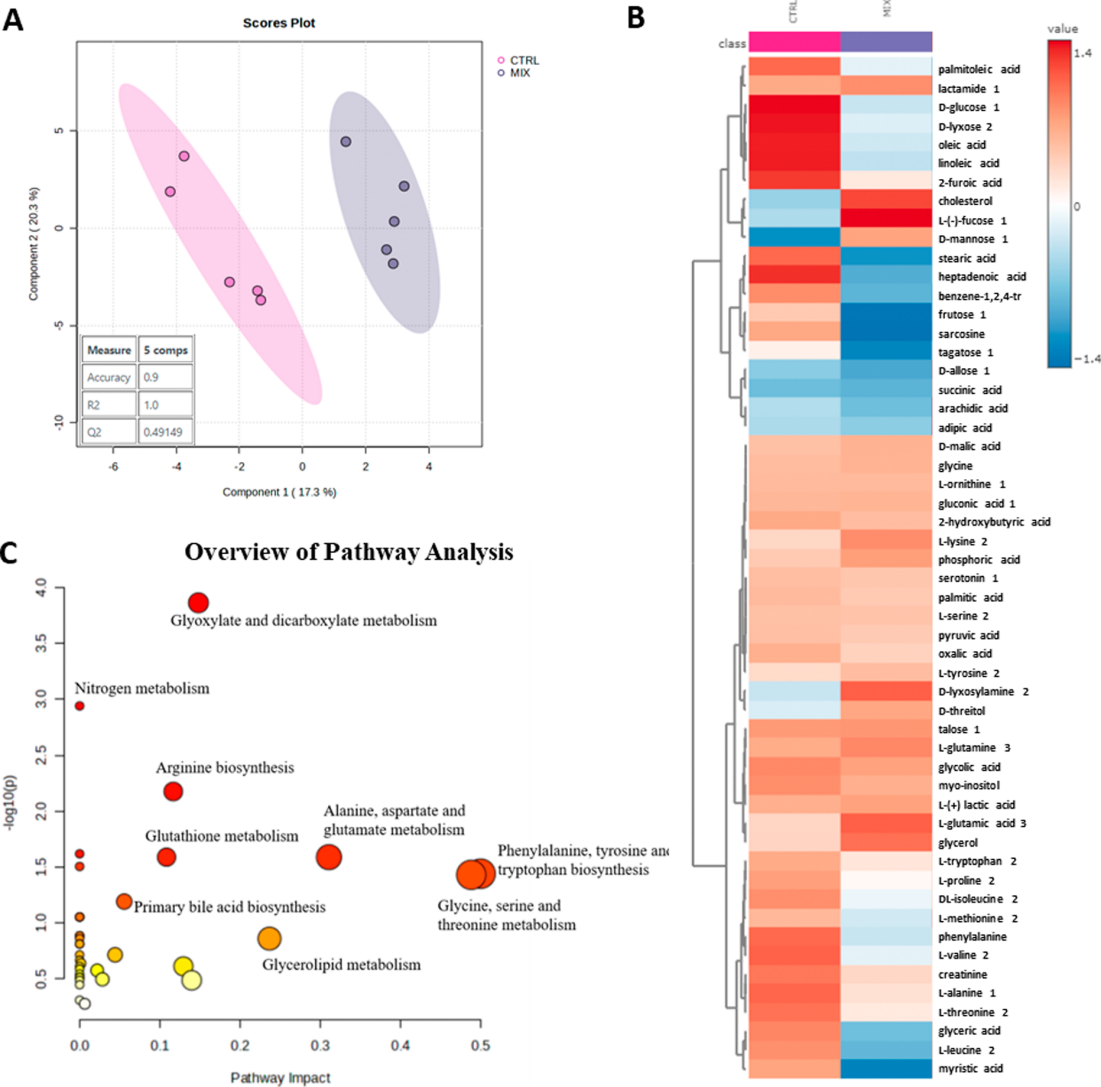
Serum metabolic perturbations induced by pesticide exposure in C57BL/6 mice. (A) PLS-DA score plot (*R* ^2^ *Y* = 1.0, *Q* ^2^ = 0.49) showing separation between CTRL (pink) and MIX (purple) groups. (B) Hierarchical clustering of significantly altered metabolites (VIP > 1.0, *p* < 0.05); red/blue indicates increased/decreased abundance versus group mean. (C) Pathway impact (circle size = pathway impact), including glutathione metabolism, glyoxylate metabolism, and amino acid metabolism. Analysis performed using MetaboAnalyst 6.0 (https://www.metaboanalyst.ca), a freely available web-based platform. Data represent mean ± SD (*n* = 5 or 6/group).

The discriminant metabolites revealed decreased levels of cholesterol, glycerol, glutamate, glutamine, l-glycine, l-serine, l-tyrosine, tagatose, d-fructose, d-lyxose, palmitic acid, oleic acid, linoleic acid, gluconic acid, and oxalic acid in the MIX-exposed group. The reduction in palmitic, oleic, and linoleic fatty acids, as indicated by the MetaboAnalyst tool for metabolic pathway analysis (Figure 2C), suggests that pesticides impaired the biosynthesis of unsaturated fatty acids. The decrease in the monosaccharides tagatose, fructose, and lyxose indicates that the metabolism of galactose, starch, sucrose, fructose, and mannose was affected by the pesticide mixture. Altered cholesterol and glycine suggest a change in the primary bile acid metabolism. Changes in l-glycine, l-serine, l-tyrosine, glutamate, and glutamine suggest that amino acid biosynthesis and metabolism were altered. The alteration in oxalic acid, glutamate, glutamine, l-serine, and l-glycine indicates that the pesticide mixture also affected glyoxylate and dicarboxylate metabolism. The glycerolipid metabolism was altered, as indicated by the increased glycerol levels. The glutathione metabolism was affected by changes in the glycine and glutamate levels.

### 3.3 Subchronic Exposure to Pesticide Mixture Causes Intestinal Dysbiosis in Mice after 6 Weeks

The microbiota was analyzed in pesticide-exposed mice compared to nonexposed mice to verify whether exposure to a mixture of pesticides could cause dysbiosis. The culturable fecal microbiota was assessed using selective and differential culture media (Figure 3). The exposed animals (MIX) showed growth of both total aerobic bacteria (Figure 3A), statistically significant, with a *p*-value of 0.0020, and total anaerobic bacteria (Figure 3B), with a *p*-value of 0.0403. For the selective media, there was an increase in the abundance of *Staphylococcus* bacteria (Figure 3C), with *p* = 0.0415. However, there was no statistical difference between the exposed and unexposed groups in the cultures for *Enterococcus* and *Streptococcus* (Figure 3D), LAB (aerobiosis), and *Bifidobacterium* + LAB (anaerobiosis) are shown in Figure 3E,F, respectively. There was no bacterial growth on BBE agar media added with 0.1% v/v gentamicin for the isolation of *Bacteroides*, nor on MacConkey agar for the isolation of *Enterobacteriaceae*.

**Figure 3:**
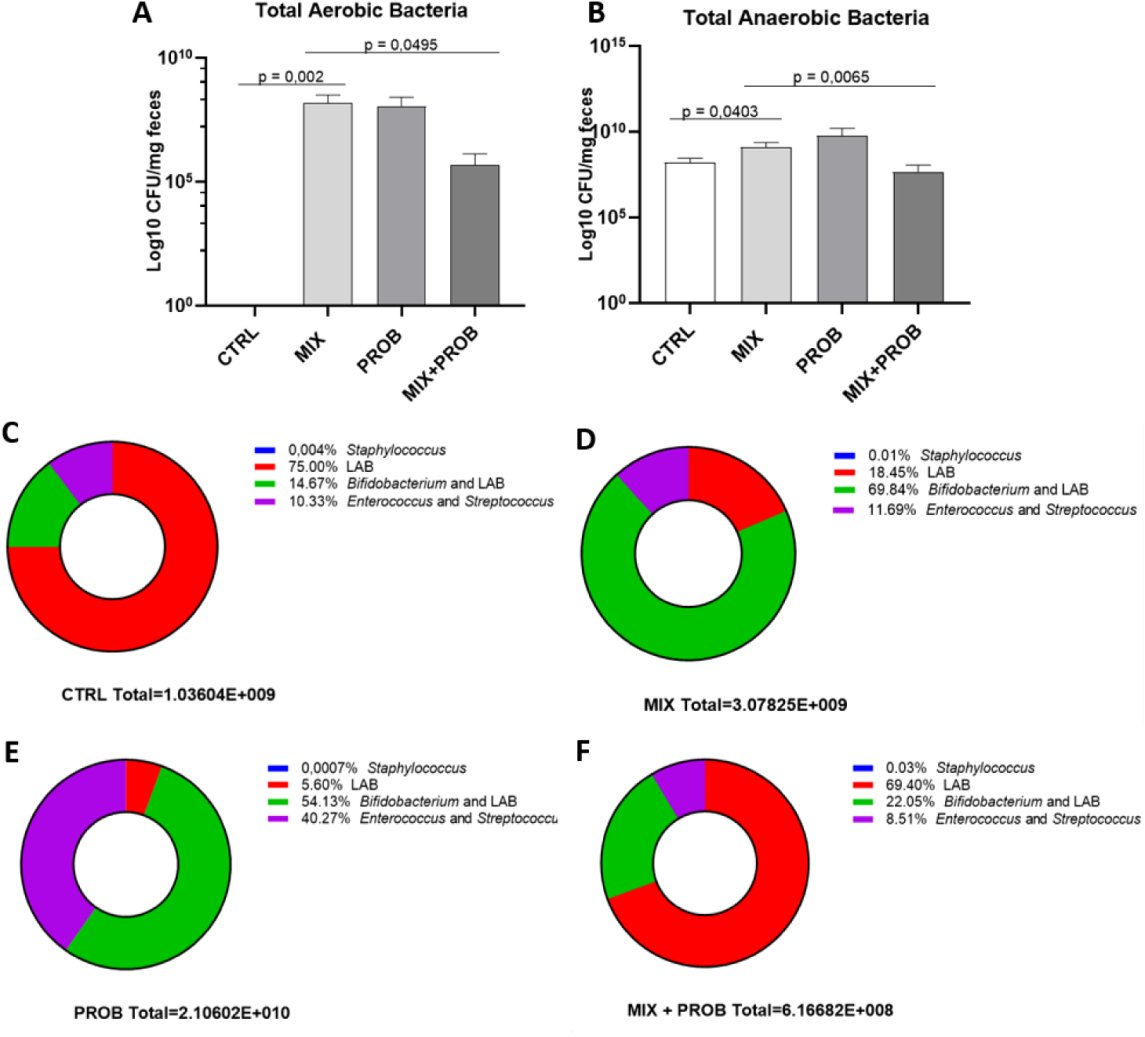
Cultivable fecal microbiota alterations in C57BL/6 mice after 6-week pesticide exposure and *B. longum* 5^1A^ intervention. (A) Total aerobic microbiota (Blood Agar, CFU/g feces). (B) Total anaerobic microbiota (Blood Agar +1% hemin/menadione and CFU/g feces). (C–F) Selective quantification of: (C) *Staphylococcus* *spp.* (Mannitol Salt Agar), (D) *Streptococcus*/*Enterococcus* *spp.* (Bile Esculin Agar), (E) aerobic lactic acid bacteria (LAB; MRS agar), and (F) anaerobic *Bifidobacterium*/LAB (MRS agar). Groups: CTRL (vehicle control), MIX (GLY-IMI-TEB mixture), PROB (*B. longum* 5^1A^), and MIX+PROB (cotreatment). Data represent mean ± SEM (*n* = 5–6/group); *p* < 0.05, Wilcoxon with Dunn correction (post hoc).

### 3.4 Subchronic Exposure to Pesticide Mixture alters Short Chain Fatty Acid Production in Mice after 6 Weeks

We evaluated the production of SCFA by the animal’s intestinal microbiota (Figure 4B,C). The analysis showed that acetate production increased by intestinal bacteria in the exposed group (9.937 ± 1.842 mmol/mg feces) compared to the control group (7.868 ± 4.468), with *p* = 0.0381, however, not in butyrate production (CTRL: 2.600 ± 1.092; MIX: 3.753 ± 1.219 mmol/mg feces, *p* = 0.0823).

**Figure 4:**
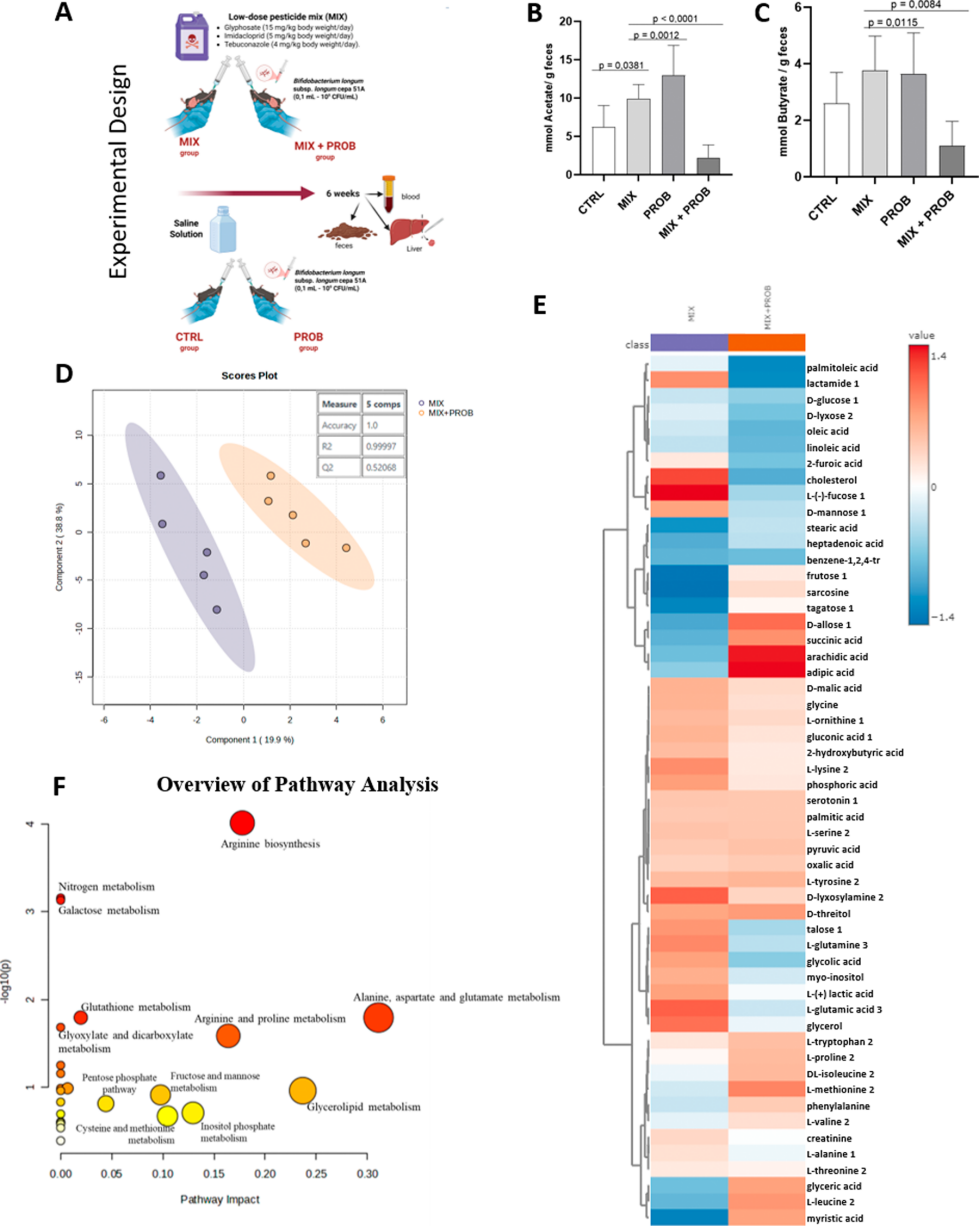
Systemic metabolic modulation by pesticide exposure and *B. longum* 5^1A^ intervention in C57BL/6 mice. (A) Experimental timeline: control (CTRL), probiotic (PROB), pesticide mixture (MIX), and pesticide + probiotic (MIX+PROB) groups. (B) Fecal acetate and (C) butyrate levels (mmol/g feces). (D) PLS-DA score plot (*R* ^2^ = 0.99, *Q* ^2^ = 0.52) showing group clustering, MIX (purple), and MIX+PROB (orange). (E) Hierarchical clustering of significantly altered metabolites (VIP > 1.0, *p* < 0.05); red/blue indicates increased/decreased abundance versus group mean. (F) Pathway impact analysis (circle size = pathway impact) including: amino acid metabolism and carbohydrate metabolism. Metabolomic analysis performed using MetaboAnalyst 6.0 (https://www.metaboanalyst.ca), a freely available web-based platform. Data represent mean ± SD (*n* = 5 or 6/group); *p < 0.05 (Wilcoxon test with Dunn’s post hoc correction).

### 3.5 Treatment with the probiotic *B. longum* 5^1A^ prevents Oxidative Stress Caused by Exposure to Pesticide Mixture in Mice

The intestinal microbiota can be modulated by probiotics, which help the host to reestablish homeostasis after dysbiosis.^25^ The mixture of pesticides altered the growth of the probiotic (Figure 3A) when we visualized the cultures of total anaerobic bacteria (in blood agar supplemented with hemin and menadione 1% v/v), with a statistically significant reduction (*p* = 0.0065, comparing the MIX and MIX+PROB groups). When specifically observing the count of anaerobic bacteria *Bifidobacterium* and LAB (Figure 3F), in anaerobiosis, we identified a tendency to reduce the count of these bacteria in the presence of the pesticide mixture (*p* = 0.0693, comparing the MIX and MIX+PROB groups).

When evaluating the SCFA production, metabolites produced by bacteria through the fermentation of dietary fiber, and the acetate was produced in more significant amounts by *B. longum* 5^1A^ as expected (*p* = 0.0065, comparing the CTRL and PROB groups). However, the production of acetate and butyrate by the probiotic was reduced in the presence of a mixture of pesticides (acetate with *p*-value 0.0044; butyrate with *p*-value 0.0084, comparing the MIX and MIX+PROB groups).

*B. longum* 5^1A^ did not change the parameters of body weight, the relative weight of organs (liver, spleen, kidneys) and periuterine adipose tissue, fasting blood glucose, insulin sensitivity, total cholesterol and triglycerides, and histopathology of organs (large intestine, kidney, spleen) (data not shown). The spleens of animals that received the treatment (PROB and MIX+PROB) were smaller than those in the CTRL group (*p* = 0.0376 and *p* = 0.0074, respectively). However, there was no statistical difference compared to the group that received the pesticide mixture (MIX), which also had a reduced spleen size.

However, when evaluating oxidative stress, the probiotic reduced lipid peroxidation caused by exposure to the mixture of pesticides, and the quantification of MDA and hydroperoxides was statistically significant (*p* = 0.0192 and *p* = 0.0392, respectively), making the levels of both markers as low as those in treatment with the probiotic without the exposure. Consequently, the genotoxic effect of pesticide exposure was mitigated, with a significant reduction in the frequency of MN (*p* = 0.0005), also making the count equal to the probiotic levels without exposure.

### 3.6 Treatment with the probiotic *B. longum* 5^1A^ Prevents Changes in the Metabolism of Mice Exposed to the Pesticide Mixture after 6 Weeks

A metabolome analysis was performed between the MIX and MIX+PROB groups to evaluate the modulation of the intestinal microbiota by *B. longum* 5^1A^ and its ability to assist in eliminating pesticides from the mixture and recovering homeostasis in exposed animals. Figure 4D illustrates the supervised separation of data from animals exposed and not exposed to the pesticide mixture (PLS-DA model parameters: *Q* ^2^ = 0.521; *R* ^2^ = 0.99997; Accuracy = 1.0). The metabolic profiles were differentiated mainly by the gluconic acid, l-glutamine, malic acid, and 2-hydroxybutyric acid levels.

Treatment with probiotics in animals exposed to the pesticide mixture identified possible altered metabolic pathways, including glyoxylate and dicarboxylate metabolism, the pentose phosphate pathway, arginine biosynthesis, nitrogen metabolism, alanine, aspartate, and glutamate metabolism, pyrimidine metabolism, purine metabolism, glutathione metabolism, arginine and proline metabolism, propanoate metabolism, and fatty acid metabolism. These changes can be visualized in Figure 4F through the pathway analysis overview generated by MetaboAnalyst 6.0.

### 3.7 Exposure to a Mixture of Pesticides Induces Oxidative Stress in the Liver of Mice

An assessment of oxidative stress in the liver was carried out by determining TBARS, represented by MDA and hydroperoxides (Figure 5B,C). There was no statistical difference between MDA concentrations in the MIX group (1.420 ± 0.636 μmol MDA/mg of protein) against the CTRL group (1.250 ± 0.221 μmol/mg of protein). The dosage of hydroperoxides showed a statistical difference (*p* < 0.05), with an increase of approximately 25% in the formation of hydroperoxides between the CTRL and MIX groups, indicating that the mixture of pesticides caused lipid peroxidation.

**Figure 5:**
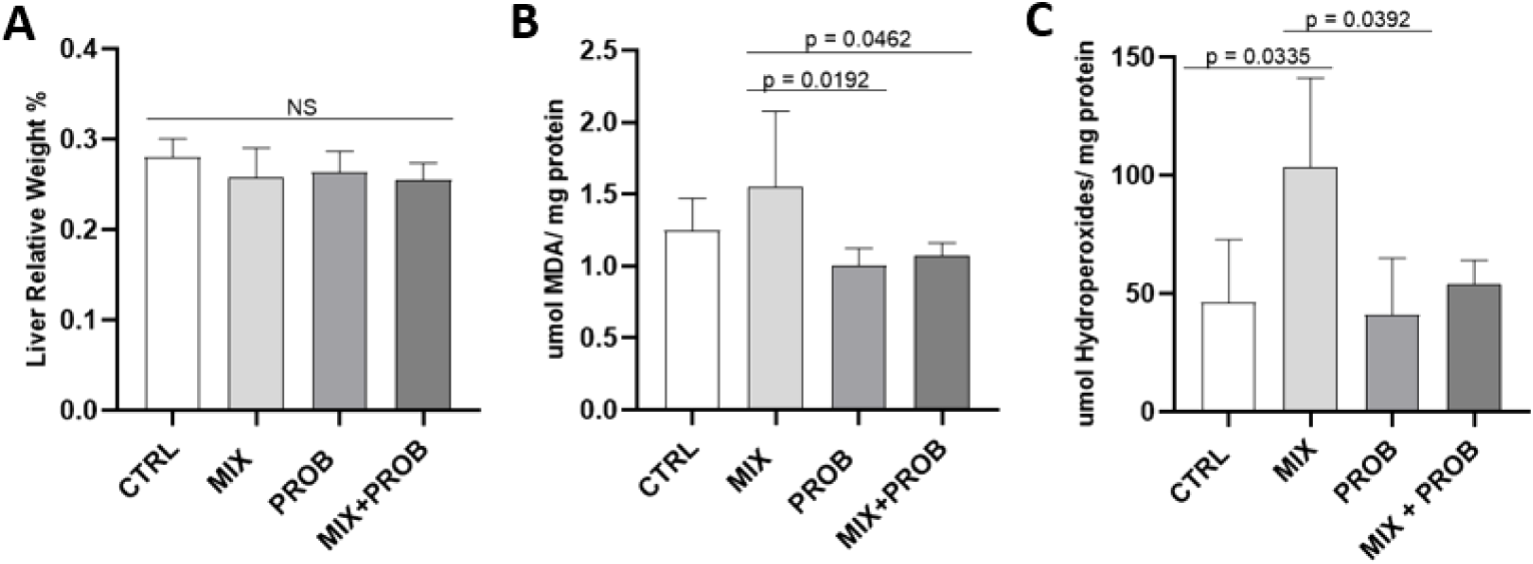
Protective effects of *B. longum* 5^1A^ against pesticide-induced oxidative damage in C57BL/6 mice. (A) Liver-to-body weight ratio (%) across experimental groups. (B) Hepatic malondialdehyde (MDA) levels. (C) Hepatic hydroperoxide concentrations. Groups: CTRL (vehicle control), MIX (GLY-IMI-TEB mix), PROB (*B. longum* 5^1A^), MIX+PROB (cotreatment). Data represent mean ± SD (*n* = 6/group); NS = not significant (*p* ≥ 0.05, Wilcoxon with Dunn correction (post hoc)).

### 3.8 Exposure to a Mixture of Pesticides Induces Micronucleus Development in the Peripheral Blood of Mice

On slides stained with Rapid Panotic (Figure 6), we can see the MN (arrow) in Figure 6A at 400× magnification, stained in dark blue, with rounded, nonrefringent shapes, within erythrocytes with intact cytoplasm. In Figure 6D (MIX), to illustrate, 3 MNs were not counted because they did not meet the necessary evaluation requirements.

**Figure 6:**
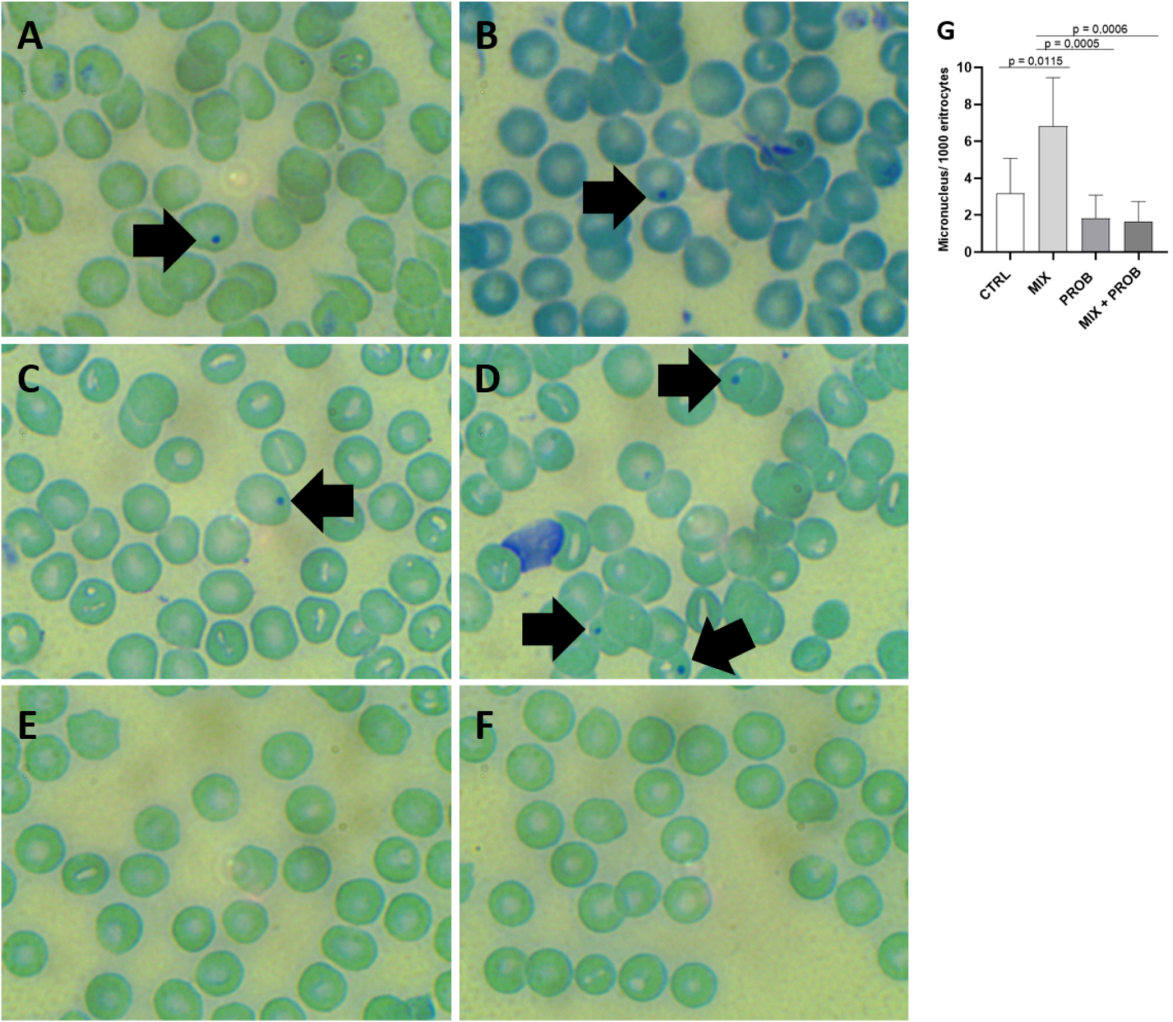
Genotoxicity assessment in C57BL/6 mice following pesticide exposure and *B. longum* 5^1A^ intervention. (A–F) Representative peripheral blood erythrocytes (Rapid Panotic stain, 400×) from (A) CTRL (vehicle control), (B) PROB (*B. longum* 5^1A^), (C–D) MIX (GLY-IMI-TEB mix), and (E,F) MIX+PROB (cotreatment). Arrows indicate micronuclei (MN, dark blue, spherical inclusions within intact erythrocytes). (G) Quantification of MN frequency per 1,000 erythrocytes. Data represent mean ± SD (*n* = 6/group); *p* < 0.05, Wilcoxon with Dunn correction (post hoc).

The frequency of MN is used as a biomarker of effect to detect mutations caused by the loss of chromatin due to structural chromosomal damage or in the mitotic process. Exposed animals (MIX) showed an increase in the frequency of micronuclei (6.83 ± 2.62 MN/1000 erythrocytes) compared to the CTRL group (3.16 ± 1.91 MN/1000 erythrocytes), with *p* = 0.0115 (Figure 6G).

## 4 Discussion

The action of pesticides as endocrine disruptors is silent but insidious, often complicating the assessment of their involvement in observed metabolic changes within an organism. In this study, we demonstrated that a mixture of three pesticides—GLY, IMI, and TEB—at low doses (below the NOAEL) caused intestinal dysbiosis, altered insulin sensitivity, increased serum total cholesterol, and decreased serum triglyceride levels in C57BL/6 mice during 6 weeks of oral (subchronic) exposure. Exposure also induced oxidative stress by increasing lipid peroxidation and exhibited genotoxicity by elevating micronuclei frequency in the mice’s peripheral blood. While treatment with the probiotic *B. longum* 5^1A^ did not reverse all subclinical changes caused by the pesticide mixture, it was critical in mitigating cellular damage and reducing oxidative stress and genotoxicity.

Our initial evaluation of body weight and relative organ weight revealed no statistically significant differences between exposed and unexposed animals. Regarding the biochemical parameters, we observed a subclinical alteration: an increase in the total cholesterol and a decrease in serum triglycerides. Fasting blood glucose levels also remained statistically unchanged. However, insulin resistance was clearly identified in the exposed animals (Figure 1C), indicating a potential progression to a diabetic state with extended exposure. The stability of fasting blood glucose, despite evident insulin resistance, is a crucial finding that highlights the subclinical stage of metabolic dysfunction. This condition, where insulin effectiveness is diminished but glucose levels are maintained by compensatory production, characterizes the initial phases of insulin resistance, preceding pancreatic exhaustion and overt fasting hyperglycemia. This suggests that a longer exposure duration or higher doses might lead to manifesting diabetes, reinforcing the insidious nature of endocrine disruption.

The absence of statistically significant alterations in body weight and periuterine white adipose tissue relative weight (Figure 1A,B), despite the observed metabolic disruptions, can be attributed to the sub-NOAEL dose and the subchronic (6 weeks) duration of exposure. Although the lack of weight change suggests no acute toxicity or overt distress, the detection of early biochemical (cholesterol, triglycerides) and metabolic (insulin resistance) alterations indicates that the pesticides are exerting insidious effects at molecular and cellular levels prior to manifesting as macroscopic changes or shifts in energy balance. This highlights the complexity of identifying impacts from low-dose, subchronic exposures, where effects can be subtle and progressive. While isolated GLY, IMI, and TEB pesticides may independently alter animal body weight, serum lipid levels, blood glucose, and insulin sensitivity,^11,45–47^ our findings suggest an additive or synergistic endocrine-disrupting effect when these pesticides are combined, resulting in altered insulin sensitivity, increased total cholesterol, and decreased triglyceride levels.

It is also noteworthy that studies on pesticide mixtures have indicated sex-specific responses, wherein male mice may exhibit weight gain due to fat accumulation, whereas female mice often do not, underscoring the importance of considering sexual dimorphism in toxicological assessments.^48,66^ Our exclusive use of female mice in this study, coupled with the observed absence of weight changes, aligns with such findings, suggesting that the initial metabolic disruptions may precede overt alterations in body composition in this specific sex.

A prior study on pesticide mixtures indicated that female mice can exhibit alterations in intestinal microbiota, characterized by increased acetate and medium-chain fatty acid production, which are often associated with intestinal diseases.^48^ Consistent with this, our study found dysbiosis in female mice, evidenced by an increase in the number of total anaerobic bacteria and acetate production. Exposure to the pesticide mixture initially prompted a change in the metabolism of intestinal microbiota, potentially impeding butyrate production. This SCFA is crucial as an energy substrate for colonocytes, activating PPARγ and consequently β-oxidation.^49,50^ A reduction in butyrate can lead to O_2_ release into the intestinal lumen, favoring facultative anaerobic bacteria. While butyrate production did not reach statistical significance (*p* = 0.0823), the observed trend of increase in the MIX group (Figure 4C) is biologically relevant and aligns with the plasticity of the gut microbiota. This trend may reflect an adaptive response where bacteria, in an effort to restore homeostasis and provide an energy substrate, increase their production of butyrate. However, continued exposure to the pesticide mixture hindered complete reestablishment of homeostasis, ultimately leading to dysbiosis and metabolic disturbances in the exposed mice.

The absence of *Bacteroides* growth on BBE agar and *Enterobacteriaceae* growth on MacConkey agar (Section 3.3) warrants further consideration. Although pesticide exposure induced dysbiosis, characterized by an increase in total aerobic and anaerobic bacteria and *Staphylococcus*, the nondetection of *Enterobacteriaceae* suggests that, under the given exposure conditions (sub-NOAEL doses and 6 weeks), the dysbiosis may be at a stage where there is no substantial proliferation of specific Gram-negative bacteria typically associated with more severe or inflammatory intestinal dysfunctions. Similarly, the absence of *Bacteroides*, a prominent and metabolically active genus in the gut microbiota, could indicate a selective impact of the pesticide mixture on the microbial community composition or that their populations remained below the detection limits of culture methods under these specific conditions.

While exposure biomarkers are commonly used to monitor environmental pollutants, effect biomarkers—such as those for oxidative stress and genotoxicity—are crucial for assessing cellular and genetic damage. The liver plays a key role in this context, as it is the primary organ responsible for detoxifying chemical compounds through biotransformation. Liver damage can disrupt systemic homeostasis, leading to adverse effects such as lipid peroxidation.^11^

The mixture used in this study induced lipid peroxidation in liver cells, as evidenced by elevated hydroperoxide levels. Previous studies have shown that the three individual pesticides can trigger oxidative stress, resulting in lipid peroxidation and disruption of the body’s antioxidant defense system. Moreover, exposure to the pesticide mixture significantly increased the frequency of micronuclei (MN) in peripheral blood erythrocytes (Figure 6G). MN frequency—a well-established biomarker for chromosomal damage and mitotic errors—exhibits a dose-dependent response, further supporting the genotoxic effects observed.^7,14,51^ Most studies correlate lipid peroxidation with elevated MN frequency following pesticide exposure.^51–54^ The observed lipid peroxidation indicates a saturation of the mice’s antioxidant defense system, permitting reactive oxygen species (ROS) to induce oxidative damage—including lipid oxidation and DNA lesions—thereby promoting micronucleus formation. Previous work confirms that these three pesticides and their metabolites stimulate ROS generation, leading to malondialdehyde (MDA) production as a marker of lipid peroxidation. Their effects primarily target mitochondrial and endoplasmic reticulum function, deplete NADPH/NADH reserves, and disrupt glutamate homeostasis in photorespiration.^5,6,55–61^ Moreover, pesticide metabolites are excreted via bile and feces, reentering systemic circulation through the enterohepatic cycle. Notably, emerging evidence links exposure to these compounds with intestinal dysbiosis, as reported in multiple studies.^62–64^

Expanding on these findings, prior research has elucidated distinct toxicological mechanisms for each pesticide. IMI undergoes extensive hepatic metabolism, with its metabolites demonstrating clastogenic potential through increased MN frequency and DNA damage.^7,65^ TEB biotransformation yields reactive metabolites that upregulate cytochrome P450 (CYP) enzymes and mediate genotoxicity via ROS-dependent pathways, ultimately disrupting mitochondrial and endoplasmic reticulum function and triggering apoptosis.^62,66^ While GLY is primarily metabolized to aminomethylphosphonic acid (AMPA), intestinal microbiota can additionally convert it to glyoxylate—a compound associated with oxidative stress and hepatic lipidosis.^58,67^ The synergistic/additive effects observed in our study likely result from the convergence of these pathways, collectively overwhelming antioxidant defenses and amplifying cellular injury.

Metabolomic analysis offers a powerful approach to evaluate the mechanistic effects of complex environmental exposures such as pesticide mixtures by capturing the dynamic interplay between genetic and environmental factors. While comprehensive metabolome studies in this context remain limited, our work demonstrates that exposure to GLY, IMI, and TEB significantly disrupts multiple metabolic pathways, including: (1) energy metabolism, (2) amino acid and lipid biosynthesis/metabolism, (3) glyoxylate metabolism, and (4) glutathione metabolism. These findings are consistent with the established literature showing that GLY interferes with galactose, purine, and urea metabolic pathways;^30^ IMI alters glycolysis, gluconeogenesis, and both amino acid and glutathione metabolism;^28^ while other pesticides similarly impact the TCA cycle, pentose phosphate pathway, alanine-aspartate-glutamate metabolism, and fatty acid biosynthesis.^28–32,48,67–72^

Mechanistic studies reveal that GLY metabolism generates glyoxylate, a metabolite with dual pathological effects: (1) inhibition of fatty acid oxidation leading to hepatic lipidosis and (2) exacerbation of oxidative stress. This process establishes a self-amplifying cycle in which oxidative-stress-induced glyoxyl formation undergoes glutathione-mediated conversion to additional glyoxylate. The resulting positive feedback loop within the glyoxylate pathway perpetuates lipid peroxidation and potentiates cellular damage.^65^

Comprehensive metabolic pathway analysis reveals that the GLY-IMI-TEB mixture induces toxicity through multiple interconnected mechanisms, with oxidative stress and mitochondrial dysfunction as the primary drivers of energy metabolism disruption. Notably, pesticide-induced gut dysbiosis elevates bacterial acetate production, which exerts systemic metabolic effects by (1) inhibiting the TCA cycle, purine metabolism, and pentose phosphate pathway, while (2) stimulating glycerol metabolism, gluconeogenesis, and fatty acid biosynthesis.^73^ Hepatic processing of this acetate amplifies its metabolic impact—conversion to acetyl-CoA fuels both the mevalonate pathway (increasing mitochondrial cholesterol synthesis) and ketogenesis.^74^ This mechanistic framework explains our key observations of elevated cholesterol levels and modified insulin sensitivity following mixture exposure.

While microbial xenobiotic elimination—particularly by probiotics—has been widely documented,^75,76^ we demonstrate for the first time the protective capacity of *B. longum* 5^1A^ against pesticide-induced toxicity. Although this strain could not completely metabolize the pesticide mixture or normalize all metabolic parameters (including total cholesterol, triglycerides, and insulin sensitivity), it significantly attenuated hepatic oxidative stress and genotoxicity. Notably, *B. longum* 5^1A^ reduced MDA levels, hydroperoxide concentrations, and MN frequency despite impaired probiotic growth and diminished SCFA production (acetate and butyrate) under pesticide exposure. This SCFA-independent protection was further elucidated through metabolomic analysis, which revealed alternative mechanistic pathways underlying the probiotic’s beneficial effects.

Genomic characterization of *B. longum* 5^1A^ by da Silva et al.^18^ identified 26 unique genes, including six specifically involved in carbohydrate metabolism. These encode enzymes for sugar transport systems and the conversion of arabinose and lyxose to xylulose-5-phosphate—a key intermediate in the pentose phosphate pathway. This genetic repertoire not only enhances the probiotic’s metabolic versatility but also provides host benefits through antiinflammatory activity and intestinal barrier protection. Importantly, these adaptive metabolic capabilities likely enable *B. longum* 5^1A^ to maintain its antioxidant functions, even under pesticide-induced stress, as observed in our study.

Although *B. longum* 5^1A^ demonstrated significant efficacy in reducing oxidative stress and genotoxicity, it failed to completely restore pesticide-induced metabolic dysregulation. This limitation primarily stemmed from the mixture’s direct inhibitory effects on probiotic viability and function, as evidenced by (1) significantly impaired growth (*p* < 0.05, total anaerobic bacteria), (2) reduced SCFA production (acetate and butyrate), and (3) declining trends in *Bifidobacterium* and LAB populations (Section 3.5). These findings suggest that xenobiotic-compromised intestinal conditions fundamentally limit the probiotic’s ability to achieve complete metabolic restoration, as optimal host modulation requires robust microbial proliferation and metabolic activity.

The multifaceted toxicity mechanisms of the pesticide mixture—spanning gut dysbiosis, oxidative stress, genotoxicity, and broad metabolic disruption (affecting carbohydrate, amino acid, lipid, glyoxylate, and glutathione pathways)—probably surpassed the restorative capacity of a single probiotic strain. While *B. longum* 5^1A^ effectively attenuated oxidative stress and genotoxicity, potentially through prioritized carbohydrate metabolism and sustained bacterial antioxidant enzyme activity, its capacity for systemic metabolic modulation appeared limited. We hypothesize that the strain’s metabolic resources were preferentially allocated to combat acute oxidative damage, thereby reducing its ability to concurrently address other metabolic disturbances.

The partial restoration of metabolic homeostasis observed in our study may reflect limitations in either the 6-week treatment duration or the administered probiotic dose, particularly given the continuous nature of pesticide exposure. Future investigations should evaluate: (1) extended treatment periods, (2) optimized dosing regimens, and (3) synergistic probiotic combinations targeting multiple toxicity pathways simultaneously. Such approaches may enhance the capacity to counteract complex pesticide-induced metabolic disturbances.

*B. longum* 5^1A^ maintains its antioxidant capacity through prioritized carbohydrate metabolism, even under pesticide-impaired acetate production, by sustaining bacterial enzymatic activity that reduces the level of ROS generation and glutathione depletion. This metabolic strategy enhances regulation of the pentose phosphate pathway, promoting NADPH production, which subsequently supports energy metabolism and fatty acid biosynthesis. Our metabolomic analysis revealed decreased glutamine (a glutathione precursor) and malate levels in treated animals, suggesting probiotic-mediated modulation of TCA cycle dynamics that optimizes energy production while minimizing oxidative byproducts.

These findings align with established probiotic antioxidant mechanisms^77^ and are further supported by two key observations: (1) the glutathione demand signature indicated by elevated 2-hydroxybutyrate (2-HB)—a known biomarker of both oxidative stress and insulin resistance, and (2) the significant 2-HB reduction in probiotic-treated animals, demonstrating improved metabolic homeostasis. The temporal nature of these effects suggests that extended treatment duration beyond 6 weeks might lead to full restoration of insulin sensitivity.

While our study incorporated rigorous experimental design and multiomics approaches (including comprehensive metabolomics), we recognize that the sample size (*n* = 6 per group) may limit the detection of subtle effects and broader generalizability. Nevertheless, the consistency of statistically significant (*p* < 0.05) and biologically meaningful alterations across multiple endpoints—including insulin resistance, lipid metabolism, oxidative stress markers, genotoxicity parameters, and distinct metabolomic profiles—provides compelling evidence for both the disruptive effects of chronic low-dose pesticide exposure and the partial protective efficacy of *B. longum* 5^1A^. The robustness of these findings despite the moderate sample size highlights the potency of the pesticide mixture’s effects. Expanded studies with larger cohorts are warranted to validate these observations and investigate the additional metabolic consequences.

## 5 Conclusions

Our study demonstrates that subchronic exposure to an environmentally relevant mixture of GLY, IMI, and TEB—each at doses below their individual NOAELs—induces endocrine disruption and metabolic dysfunction in C57BL/6 mice. Key manifestations included dyslipidemia, insulin resistance, and gut dysbiosis characterized by facultative anaerobe dominance and impaired acetate production. Mechanistically, these effects arose through synergistic actions: (1) oxidative stress-mediated damage to hepatic and metabolic pathways and (2) genotoxic effects confirmed by MN assay. Metabolomic profiling revealed central disruptions in the pentose phosphate pathway (NADPH depletion), TCA cycle (malate reduction), and glutathione metabolism, hallmarks of systemic redox imbalance.

Notably, *B. longum* 5^1A^ treatment provided significant cellular protection, reducing oxidative stress and genotoxicity, independent of SCFA production. Genomic and metabolomic evidence suggests this protection stems from the strain’s unique carbohydrate metabolism genes, which sustain NADPH production for glutathione regeneration while modulating glyoxylate detoxification. However, the probiotic’s partial restoration of metabolic parameters indicates either insufficient treatment duration or the need for multistrain interventions targeting broader pathways.

These findings carry critical regulatory implications. The observation that NOAEL-exceeding effects emerged only in mixture exposure challenges current risk assessment paradigms. We propose three essential reforms: (1) the incorporation of mixture toxicity evaluations in ADI determinations, (2) mandatory assessment of microbiome disruption in pesticide approvals, and (3) adoption of metabolomic biomarkers for cumulative risk assessment. This study underscores how low-dose chemical mixtures may threaten metabolic health through nonlinear, system-level interactions, a phenomenon urgently requiring policy attention.

## Supplementary Material



## Notes

Model robustness assessment by Random Forest analysis (Figure S1) (DOCX)

## Abbreviations

2-HB: 2-Hydroxybutyric acid
ADI: Acceptable Daily Intake
GLY: Glyphosate
IMI: Imidacloprid
MDA: Malondialdehyde
MN: Micronucleus
MRS: De Man, Rogosa, and Sharpe
NADH: Nicotinamide Adenine Dinucleotide
NADPH: Nicotinamide Adenine Dinucleotide Phosphate
NOAEL: No Observed Adverse Effect Level
PCA: Principal Component Analysis
PLS-DA: Partial Least Squares Discriminant Analysis
ROS: Reactive Oxygen Species
SCFA: Short-Chain Fatty Acids
TBARS: Thiobarbituric Acid Reactive Substance
TCA: Tricarboxylic Acid Cycle
TEB: Tebuconazole
VIP: Variable Importance in Projection.
