# Supplementary material for: Probiotic Bifidobacterium longum Subsp. longum 51A Protects Mice from Genotoxic and Metabolic Alterations Induced by Subchronic Exposure to a Low-Dose Pesticide Cocktail
Source: ACS Omega. 2025 Aug 4;10(32):36238–51. doi: 10.1021/acsomega.5c04121 (PMC12368810; doi:10.1021/acsomega.5c04121)
Supplement: Supplementary file 1 [file ao5c04121_si_001.docx]

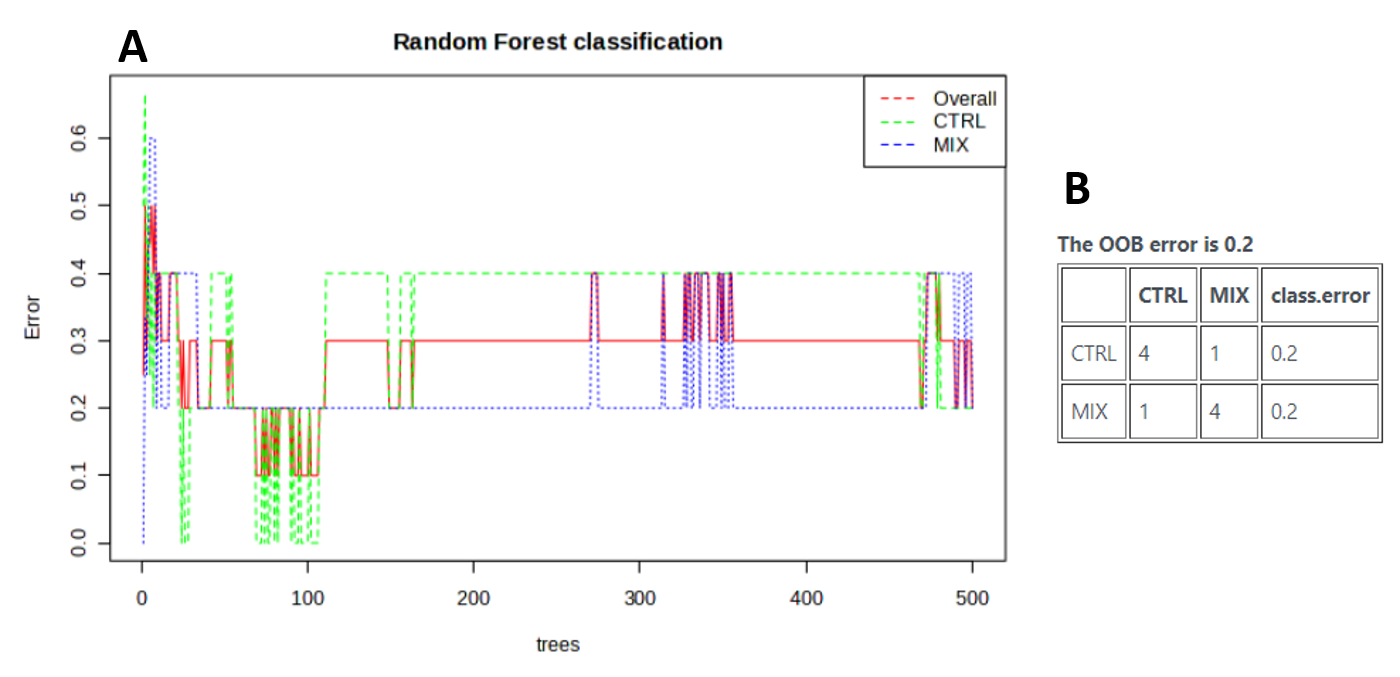


**Figure S1.** Model robustness assessment by Random Forest analysis. (A) Out-of-bag (OOB) error curve showing stabilization at 500 trees. (B) Final OOB error = 0.20. Analysis performed using MetaboAnalyst 6.0.
